# Efficacy of a certified modular ultrasound curriculum

**DOI:** 10.1007/s00101-020-00730-9

**Published:** 2020-02-13

**Authors:** R. Tomasi, K. Stark, P. Scheiermann

**Affiliations:** Department of Anesthesiology, University Hospital, LMU Munich, Marchioninistr. 15, 81377 Munich, Germany

**Keywords:** Ultrasound in anesthesia, Modular ultrasound curriculum, Certified ultrasound course, Ultrasound teaching, AFS curriculum, Ultraschall in der Anästhesie, Modulares Ultraschall-Kurssystem, Zertifizierter Ultraschallkurs, Ultraschalllehre, AFS-Seminarreihe

## Abstract

**Background:**

In recent years, ultrasound (US) has become more incorporated into anesthesia and intensive care medicine. The German Anesthesia Society established a modular curriculum to teach US skills. Until now, the efficacy of this modular curriculum has not been validated.

**Objective:**

The main objective of this study was to determine whether there is an increase of knowledge and of psychomotor skills for the trainees in this curriculum.

**Material and methods:**

After ethical committee approval, 41 anesthesia physicians were enrolled. To determine the increase of knowledge and of practical skills theoretical and practical tests performed were evaluated before and after two different US courses.

**Results:**

Comparing before and after course tests, the participants showed significant improvement in theoretical multiple choice tests (*p* = 0.008). Regarding psychomotor skills following course 1, the trainees improved significantly in the time needed to perform the two practical tests (*p* = 0.03), but not in the performance of the test. Better needle visualization during simulated US-guided vessel puncture (*p* = 0.52) and better identification of the anatomical structures in the axillary region (*p* = 0.56) could not be achieved.

**Conclusion:**

This study shows that although this US course curriculum has positively enhanced the trainees’ theoretical knowledge of US practice, it does not enhance the practical application of that theoretical knowledge. To improve this curriculum, a supervised clinically practical training should follow the course.

**Electronic supplementary material:**

The online version of this article (10.1007/s00101-020-00730-9) provides the two questionnaires in German. The article and additional material are available at www.springermedizin.de. Please enter the title of the article in the search field, the additional material can be found under “Ergänzende Inhalte”.

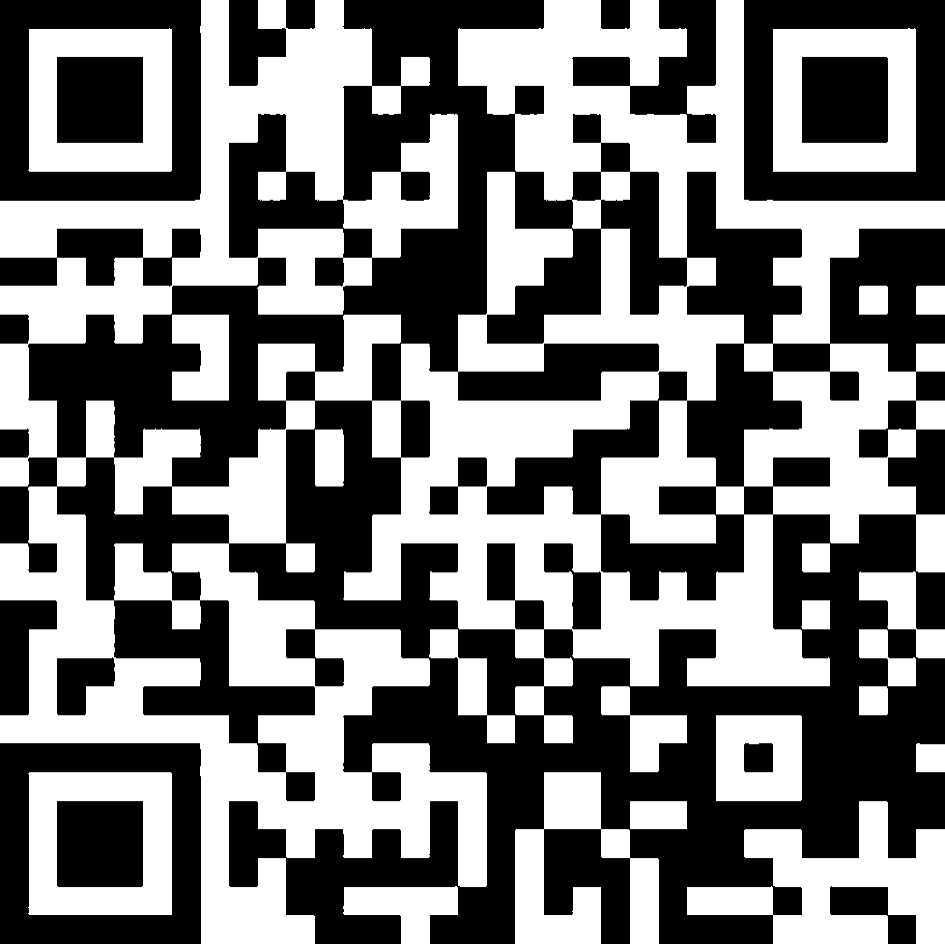

## Zusammenfassung

**Hintergrund:**

Ultraschall (US) hat in den letzten Jahren zunehmend Einzug in die Anästhesie und Intensivmedizin erhalten. Die Deutsche Gesellschaft für Anästhesiologie & Intensivmedizin hat ein modulares Kurssystem zur Vermittlung von US-Kompetenzen entwickelt. Die Wirksamkeit dieses modularen Curriculums wurde bisher nicht validiert.

**Ziel:**

Ziel dieser Studie war zu ermitteln, ob es bei den Teilnehmern durch den Besuch von Modulen dieses Kurssystems zu einer Verbesserung theoretischer Kenntnisse und praktischer Fertigkeiten kommt.

**Material und Methode:**

Nach Zustimmung durch die Ethikkommission wurden 41 Anästhesisten in die Studie eingeschlossen. Um den Zugewinn an Wissen und praktischen Fertigkeiten zu ermitteln, wurden theoretische und praktische Tests vor und nach zwei verschiedenen US-Kursen ausgewertet.

**Ergebnisse:**

Nach den Kursen zeigten die Teilnehmer eine signifikante Verbesserung in den Ergebnissen der Multiple-Choice-Tests (*p* = 0,008). Hinsichtlich der psychomotorischen Fähigkeiten nach Kurs 1 verbesserten sich die Teilnehmer in der zur Durchführung der beiden praktischen Tests benötigten Zeit signifikant (*p* = 0,03), nicht aber in der Durchführung der Tests. Die praktischen Aufgaben konnten nach dem Kurs 1 zwar signifikant schneller durchgeführt werden (*p* = 0,03), die Qualität der Nadelführung (*p* = 0,52) und die korrekte Benennung anatomischer Strukturen in der Achselregion konnten aber nicht verbessert werden.

**Schlussfolgerung:**

Diese Studie zeigt, dass obwohl dieses Kurssystem die theoretischen Kenntnisse über die US-Praxis verbessert hat, die praktische Anwendung dieses theoretischen Wissens aber nicht verbessert werden konnten. Um dieses Curriculum zu verbessern, sollte es von einem praktischen Training unter Anleitung ergänzt werden.

## Background

In recent years, ultrasound (US) has become incorporated into anesthesia and intensive care medicine because it is a useful, non-invasive, portable, and relatively low-cost diagnostic imaging method and guiding tool for procedures [3]. Healthcare providers need to be trained in order to incorporate new procedures into routine clinical practice. Psychomotor skills are best acquired using a sequenced and step-by-step teaching approach [20]. There are a variety of widely accepted and published teaching models advanced by Fitts, Simpson and Posner [21]. The number of teaching steps used in these models varies from 2 to 11 [21]. Taking the various teaching models into account, many specialized programs and workshops have been designed to teach sonographic techniques to novice operators. Until now, no program has been found to be superior to the other. American and European guidelines have been published to offer assistance in the organization of US teaching programs [27]. In addition, for critical care sonography two international expert statements have acknowledged the challenges in providing appropriate training in echography and critical care US [8, 19]. In Germany this increased use has led to the introduction of an US curriculum in teaching programs. In 2011, the German Society of Anaesthesiology and Intensive Care Medicine (DGAI) established a modular curriculum called anesthesia-focused sonography (AFS) [4, 14, 25, 28, 29]. The first module covers basic aspects of the physical principles behind US, system and transducer technology as well as principles of doppler sonography [4]. The second module deals with vascular sonographic techniques and options for further use [29] and the third module with US for regional anesthesia [14]. Module 4 focuses on transthoracic echocardiography (TTE) [28] and module 5 on the sonography of the thorax and abdomen [25]. Module 4 was revised after conducting this study in 2017 and is now integrated into a new training concept called “Perioperative fokussierte Echokardiographie in der Anästhesiologie und Intensivmedizin (PFE, perioperative focused echocardiography in anesthesiology and intensive care)”. This new curriculum consists of five modules and has a distinct theoretical and practical division [11]. The AFS curriculum of the German society is a combination of didactic lectures followed by a two-step instructional approach to teach psychomotor skills in the form of hands-on training. Following the course, practitioners receive an attendance confirmation. A certification after additional clinical teaching is not issued. There is an option for obtaining various, however not validated degrees of a certification, provided by the interdisciplinary German Society of Ultrasound in Medicine (DEGUM). To our knowledge, the efficacy of the AFS modular curriculum of the DGAI has not been validated to date. Performing US based psychomotor skills requires the operator to develop visuomotor and visuospatial skills [20]. Probably, these skills cannot be learned through hands-on training sessions only, but only in the context of everyday clinical practice. Therefore, the main objective of this study was to determine if there is a relevant increase of knowledge and of psychomotor skills for the participants of the AFS curriculum.

## Material and methods

The ethics committee of the Ludwig-Maximilians-University Munich (ethics committee number 593-16) approved the study, and participants provided written informed consent. For this monocentric prospective study 41 anesthesiologists of the University Hospital of Munich were enrolled. The anesthesiologists had different prior practical experience in sonography; the only exclusion criterion was earlier participation in certified US courses. Of these participants, 22 followed AFS modules 1–3 and 19 AFS modules 4–5. Modules 1–3 were held in a 2-day course in November 2016 (course 1), and modules 4 and 5 in a 2-day course in February 2017 (course 2). Because of the different focus of the two courses, prior participation in course 1 was not mandatory in order to attend course 2.

**Fig. 1:**
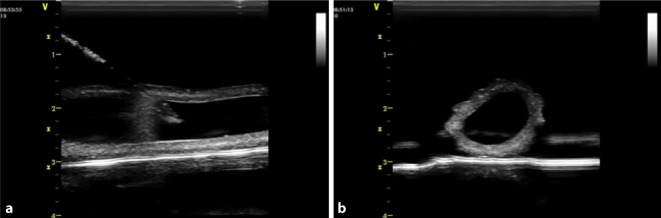
Custom made gelatine puncture model. The figure shows **a** tip of a regional anesthesia needle in a noodle in in-plane puncture technique and **b** cross-section of a noodle embedded in gelatine

To assess baseline knowledge, all participants of course 1 filled out a 15-question multiple choice test in the hospital without external sources of information 1 week before participating in the courses (the multiple choice test is provided as a supplement). In brief, the multiple choice test was created in Microsoft Word (Microsoft Corporation, Redmond, WA, USA). A maximum of 30 points could be achieved. Together with the multiple choice test, all participants performed two practical exercises to assess psychomotor skills. To test visuomotor skills, a longitudinal (in-plane) puncture technique [6] was used on a custom made puncture model (Fig. 1). The goal of the task was to penetrate a noodle (Barilla, Tortiglioni, 9 mm; Barilla, Parma, Italy) embedded in gelatine at 3 cm depth with the tip of a regional anesthesia needle (Uniplex NanoLine, Pajunk^Ⓡ^, Geisingen, Germany) using an in-plane puncture technique and to confirm that the tip of the needle is in the required position. A 38 mm 10–12 Hz linear transducer was used to visualize the real-time track of the needle to the target. An intervention imaging preset was selected which used a grey scale map with a frame rate of 24.5 frames per second and one focal zone. First, the time needed to complete the skill was measured (test 1). The time was started when the needle was picked up by the trainee and stopped when the trainee stated that the needle was inside the lumen of the noodle in the required position. This essentially mirrors the clinical use of US, as in a clinical setting there is no instructor to state whether the needle is in the correct place. It is up to the judgment of the operator and whether he considers the puncture to be successful. The time was only utilized for further analysis if the tip of the needle was correctly inside the noodle. Otherwise the task was considered incorrectly performed and this was recorded in the data. An instructor rated the task as 0 when during the entire procedure the needle tip and the shaft were never observed on two dimensional (2D) real-time visualization, as 1 when the needle shaft but not the tip was observed, as 2 when the needle tip but not the shaft was observed, and a mark of 3 was obtained when both the tip of the needle and shaft were continually observed.

To test visuospatial skills, the trainees had to demonstrate a 2D image of the axillary brachial plexus on a human model (test 2) visualizing prespecified structures. All participants performed the skill on the same human model. To obtain the best view of the brachial plexus a linear transducer was to be placed in the transverse plane at the lateral border of pectoralis major muscle. To optimize image quality, appropriate depth, focus range and gain had to be set by the operator. The structures of interest for this task were the axillary artery, the axillary veins, and the four terminal branches of the brachial plexus: the median (superficial and lateral to the artery), the ulnar (superficial and medial to the artery) and radial (posterior and lateral or medial to the artery) and the musculocutaneous (between the biceps and coracobrachialis muscles) nerves. Once the trainees were able to display all anatomical structures of interest on a 2D image, they had to print this image and correctly label the anatomical structures displayed on the printout. For each correctly identified anatomical structure, 0.5 points were allocated and a maximum of 3 points could be achieved. In addition, the time taken to create the image was measured. The time was started when the operator picked up the transducer and stopped when printing of the image was started.

To determine the increase of knowledge and of performance in psychomotor skills, the multiple choice test with identical questions in a randomized order plus the skills tests were repeated 1 week after the course. The following results were examined for each assessment: scores of the multiple choice tests, time to successfully perform test 1, the scores for needle visualization of test 1, time to successfully perform a 2D image of the axillary brachial plexus visualizing the aforementioned structures of interest, and scores of the anatomical structures visualized in the trainee made images.

For the pretest and posttest of course 2, a presentation in Microsoft PowerPoint (Microsoft Corporation) with a 20-question multiple choice test regarding the evaluation of US sequences or images were created. The questions were related to the topics covered by the AFS modules 4 and 5 and are provided as a supplement. The participants could access the computer-based diagnostic test under their clinic account, but the answers were marked on an enclosed answer sheet. Multiple answers were possible. The numbers of correct answers were not stated in the question. If the question was answered completely correct, 2 points were given, with partially correct answers being worth 1 point and 0 point for no correct answer. Consequently, a maximum of 40 points could be achieved. To assess baseline knowledge and increase of knowledge the pretest and posttest were respectively performed 1 week before and after the course.

The topics covered in course 1 allowed the participants to test their newly learned practical skills on healthy human models. Course 2 contained more pathological findings, and hence it was not possible for the participants to test their practical skills on healthy human models. During the course, hands-on training was organized in a rotation system, where trainee groups moved to the next station after a defined time frame of 25 min. Each station focused on a different task. The sessions were designed for small teaching groups with a maximum of five trainees on one instructor in order to guarantee 5min of teaching per trainee per station. The instructors did not rotate.

Statistical analysis was performed by using GraphPad Prism 5 (GraphPad Software, La Jolla, CA, USA). We evaluated the data for normal distribution with the Shapiro-Wilk test. In the case of normal distribution, the values are represented as mean (standard deviation, SD), otherwise as median (25th/75th percentile). Paired-*t*-test or the Mann-Whitney *U*-test were used to compare the values. All reported *p*-values are 2‑tailed. *P*-values <0.05 were considered statistically significant.

## Results

**Fig. 2:**
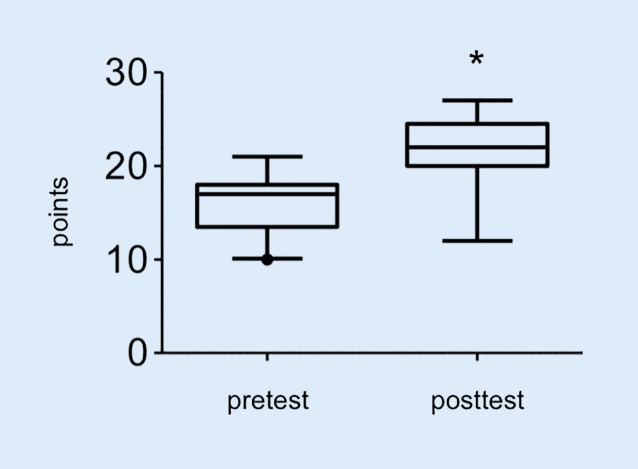
Theoretical test course 1: points achieved for the pretest and posttest (*n* = 22). *Asterisk* *p* < 0.0001

Demographic characteristics of the study participants are shown in Table 1. The trainees did not differ in age and anesthesia work experience in both courses and we found no age or work experience differences within the course groups. In course 2 two participants did not give any information about gender and age. In course 1 the participants achieved an average of 16.10 (SD 2.98) points in the multiple choice pretest, in the posttest an average of 21.86 (SD 3.68) was reached. The improvement of the score (Fig. 2) was statistically significant (*p* < 0.0001).

**Table 1:** Demographic characteristics of the study participants

	Course 1	Course 2	*p*-value
Age [years]	39 (35.5/45)	39.38 (SD 5.26)	Ns
Gender [male/female]	12/10	11/6	Ns
Work experience [years]	11 (6.87/19.25)	11.32 (SD 5.55)	Ns

Values are represented as median (25th/75th percentile) for course 1 and as mean (standard deviation, SD) for course 2

Significance for *p*-values <0.05

*SD* standard deviation

**Fig. 3:**
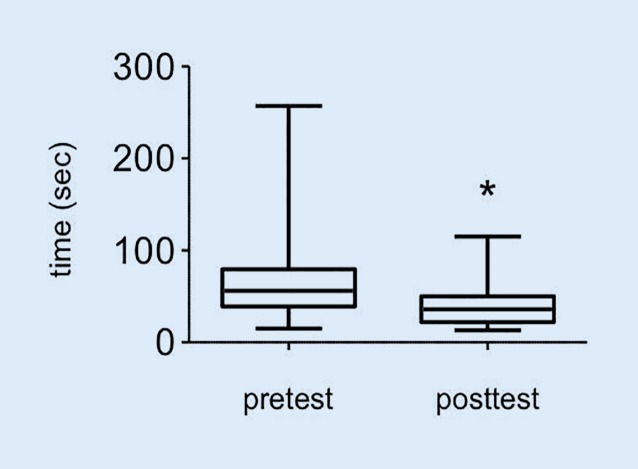
Time in seconds needed for noodle puncture. *Asterisk* *p* < 0.03

**Fig. 4:**
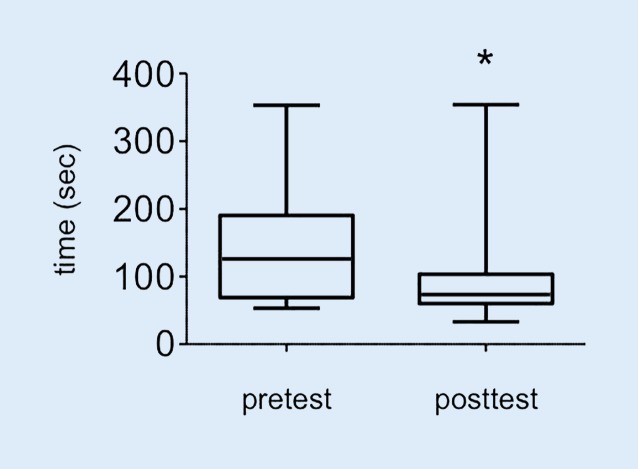
Time in seconds needed to visualize the plexus axillaries. *Asterisk* *p* < 0.03

**Fig. 5:**
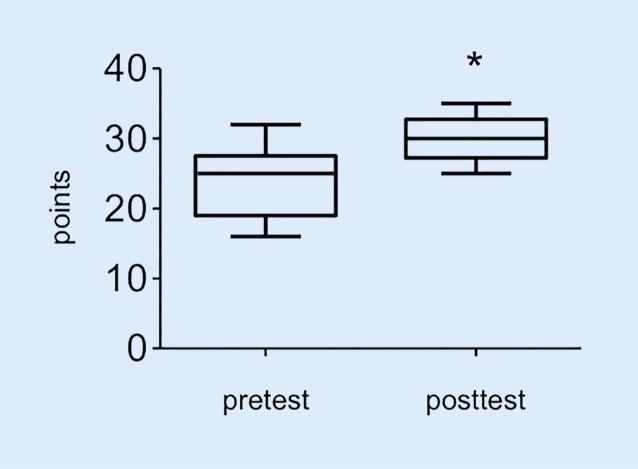
Computer-based diagnostic test points achieved for the pretest and posttest (*n* = 19). *Asterisk* *p* < 0.008

For test 1 (Fig. 3), no trainee failed to perform the task. The participants improved significantly in the time needed to complete the skill from an average of 56s (25/75 percentiles: 39/79.50s) seconds in the pretest to an average of 36s (25/75 percentile: 22/50s) seconds in the posttest (*p* = 0.03). However, the quality of the needle visualization did not improve between the pretest (2, 25/75 percentile: 2/2.5) and the posttest (2, 25/75 percentile: 2/3) (*p* = 0.52). For test 2 (Fig. 4), the time to create a 2‑dimensional image of the axillary brachial plexus improved from 140.90s (SD 77.99s) to 73.50s (25/75 percentile: 60/103.5s) seconds (*p* = 0.03). No significant improvement was achieved in the correct identification of the anatomical structures from the pretest (1.45, SD 1.01) to the posttest (1.53, SD 0.72) (*p* = 0.56). In course 2, the participants improved in the computer-based diagnostic test (Fig. 5) from an average of 23.80 (SD 4.78) points in the pretest, to an average of 29.23 (SD 3.05) points in the posttest (*p* = 0.008).

## Discussion

This study demonstrates that a modular US curriculum with didactic lectures and hands on training may be helpful in teaching US use for anesthesiologists, as trainees had significant improvement in their theoretical knowledge and in the time needed to perform psychomotor skills; however, an improvement in needle visualization in a visuomotor skill and in identification of correct anatomical structures in a visuospatial skill could not be achieved.

Sonographic techniques are challenging to learn. Learning US requires the integration of multiple skill sets including identification of appropriate patients, image acquisition, image interpretation, and integration of findings into the clinical management of patients [18]. To perform medical sonographic examinations, the use of psychomotor skills is crucial. Central components of medical US imaging are visuomotor and visuospatial psychomotor skills [20]. These skills are best acquired in stages using a sequenced and stepped teaching approach. Therefore, critical care US programs include didactic teaching, direct supervision and maintenance of a logbook [9]. Unlike other programs, the German AFS sonography curriculum includes a combination of both didactic lectures and hands-on training. An attendance confirmation is provided but without proof of clinical practical training. For this reason, we tried to show whether or not an US course without subsequent retraining in daily clinical practice improves participants’ knowledge and psychomotor skills. We were able to show that these skills can only partially be improved by the given US courses. The trainees improved significantly in the time needed to perform the tasks, but not in puncture quality and in correctly identifying anatomical structures. In contrast, theoretical knowledge improved significantly. Consequently, we do not feel that completion of this course is sufficient to perform independently US for regional anesthesia or for diagnostic purposes.

In this study, psychomotor skills were practiced by the trainee immediately after lecture demonstration. The instructors followed a traditional two-step model to teach psychomotor skills. After a short demonstration of the skill the instructor acted mainly as a coach, following instructional strategies relevant for teaching complex skills in US [18, 21]. Despite frequent hands-on training during the course, it seems likely that an improvement in psychomotor skills can only be achieved through everyday clinical practice. Indeed, a longitudinal US curriculum, compared to a single stand-alone workshop, improved the ability of internal medical residents to correctly identify static US images at 6 months [15]. In addition, after 1 year of training point of care sonography anesthesiology residents can successfully acquire images of acceptable quality [24]. Interestingly, the greatest improvements in quality and acquisition time were for vascular access and pulmonary US. In an emergency medicine study, physicians acquired the ability to interpret focused assessment with sonography in trauma (FAST) images earlier than the technical skills required to actually perform the examination. The incidence of specific technical errors improved with hands-on experience [12]. From this study it was concluded that more than 60 examinations are needed to reduce the incidence of technical errors below 5%. In contrast, for focused TTE, other studies have demonstrated that it can be performed by novice practitioners with minimal training only [13, 23]. As in our course, the goals were achieved using a combination of both didactic lectures and small group skills training, which were practiced by the learner immediately after the initial lecture demonstration to facilitate the clinical expertise and skill reinforcement [23]. In addition, an only 60-min didactic presentation on TTE for intensive care unit (ICU) trainees may be useful in teaching basic TTE skills and encouraging the use of bedside TTE in the ICU [17]. Such a short didactic presentation was chosen, because the authors proposed that one of the most common barriers to implementing TTE education is the lack of time. The barriers to delivering a high-quality training program are often the lack of trainers and no spare time for further medical training [9]. The major challenge in the posttest was to perform better in describing anatomical structures correctly and in better needle visualization in our puncture model. Although all participants have entered the noodles without continuous needle visualization thereby saving time, advancing the needle without observing the needle tip is not recommended due to a higher complication rate during vascular access procedures and also during regional anesthesia [26]. To improve these practical skills it seems mandatory, that clinical experience must be supported by a trainer and supervised by an expert and, thus, constantly mentored after an US course. Effectiveness of the educational modality used in sonographic training merits further investigation. Delivery of didactic teaching varies between face to face courses and online teaching modules, and differs in the duration and structure. Model simulation education strategies may improve training more than standardized didactic lectures. Videoclip-based pathology lectures may improve content retention [24]. These two education strategies are also used for the AFS curriculum. They could perhaps be extended by exercises on real patients in order to improve practical skills. It has been shown that education taught on non-ideal, real-world patients and not healthy volunteers can increase the efficacy of learning [17]. In addition, novices’ sonographic skills showed greater improvement when feedback was combined with validated metrics [1]. Systematic reviews have shown that simulation-based training in health care is significantly more effective than alternative teaching methods or no intervention [7]. Simulation-based medical education training was effective in improving short and long-term competency in, and knowledge of central venous catheter insertion [5]. Most likely for lack of time, the AFS curriculum does not incorporate this teaching methodology; however, the new PFE curriculum includes US simulators as an optional tool and it could be an additional methodology in the context of clinically practical training also in the other AFS modules. As an alternative to the two-step model used during the hands-on training, other step-by-step instructional models could perhaps improve the training modules. The efficacy of using different step-by-step instructional models to teach psychomotor skills is subject of ongoing debate. When using the five-step George and Doto model or the Walker and Peyton four-step model, simple skill acquisition was significantly enhanced in studies [30, 31]. In contrast, other studies comparing different skill teaching models identified no significant differences in cases of complex skill acquisition [2, 10, 16, 22]. Because of this paucity of evidence it does not seem necessary to change the use of the two-step model.

### Limitations

The study has several limitations. First, it was conducted at a single center, which limits generalizability. Moreover, additional ultrasound exposure during the follow-up period after the course was not accounted for. Finally, we did not provide long-term follow-up to assess skill retention. Nevertheless, we have for the first time scientifically assessed the efficacy of the AFS curriculum of the DGAI. We show that the AFS curriculum provides improvement in theoretical US knowledge but needs improvement in teaching practical skills.

## Conclusion

This study showed that although the AFS US course curriculum has positively enhanced the trainees’ knowledge of US practice, it did not enhance the practical application of that knowledge. The trainees could not improve their performance in the implementation of psychomotor skills. As a supplement to the AFS curriculum, a systematic practical training of sonographic techniques should follow the course. This could be provided by routine clinical work under supervision, or by special courses including simulation-based training. Further studies should aim to analyze the duration of this additional practical trainings.

## Caption Electronic Supplementary Material

ESM_Kurs 1_Fragebogen/Questionnaire (in German)

ESM_Kurs 2_Fragebogen/Questionnaire (in German)
