# Supplementary material for: Efficacy of a certified modular ultrasound curriculum
Source: Anaesthesist. 2020 Feb 13;69(3):192–7. doi: 10.1007/s00101-020-00730-9 (PMC7056694; doi:10.1007/s00101-020-00730-9)
Supplement: Supplementary file 1 — ESM_Kurs 1_Fragebogen/Questionnaire (in German) [file 101_2020_730_MOESM1_ESM.pdf]

Theoretischer Prätest

Bitte tragen sie Ihr Pseudonym ein.

Pseudonym	1☐ 2☐ 3☐ 4☐<br>1. Stelle: Erster Buchstaben des Vornamens des Vaters („B“ für Bernd)<br>2. Stelle: Erster Buchstaben des Vornamens der Mutter („H“ für Hannelore)<br>3. Stelle: Erster Buchstaben des eigenen Geburtsortes („A“ Aachen)<br>4. Stelle: Letzte Ziffer des eigenen Geburtsjahrgangs („7“ für 1977)

Es sind **Mehrfachantworten** möglich. Kreuzen Sie die richtige/n Antwort/en an (z.B. A).
Bitte verwenden Sie **keine Hilfsmittel**.

1) Bei der sonoanatomischen Darstellung der beiden Anteile des Nervus obturatorius unterhalb des Ligamentum inguinale liegen der Ramus anterior und der Ramus posterior des Nervus obturatorius in der Regel in unmittelbarer Nähe zu folgendem/n Muskel/n:

A) M. adductor longus
B) M. glutaeus maximus
C) M. adductor brevis
D) M. adductor magnus
E) M. glutaeus medius
F) M. iliopsoas

2) Welche/s Artefakt/e zeigen sich typischerweise beim Längsschnitt in der Lungensonographie?

A) Reverberationen
B) Spiegelartefakte
C) Dorsale Schallauslöschung
D) Kometenschweifartefakte
E) Randschatten – Artefakte

3) Zwischen welchen Muskeln lässt sich beim ultraschallgesteuerten interskalenären Plexus brachialis Block die typische Perlenkette darstellen?

A) M. scalenus anterior und M. scalenus posterior
B) M. scalenus medius und M. levator scapulae
C) M. scalenus medius und M. scalenus posterior
D) M. scalenus medius und M. scalenus anterior
E) M. lavator scapulae und M. Scalenus anterior

4) Unterhalb des Kniegelenks werden folgende anatomische Areale durch Äste des Nervus ischiadicus sensibel innerviert:

A) Dorsaler Unterschenkel (Nervus cutaneus surae medialis)
B) Medialer Unterschenkel (Rami cutanei cruris mediales)
C) Lateraler Unterschenkel (Nervus cutaneus surae lateralis)
D) Fußsohle (Nervus plantaris medialis)
E) Ferse (Nervus suralis)

5) Welche der folgenden Aussagen ist/sind **falsch**?

A) Beim TAP Block wird das Lokalanästhetikum zwischen dem Musculus obliquus internus abdominis und dem Musculus transversus abdominis appliziert.

B) Beim TAP Block wird auch das Peritoneum viszerale anästhesiert.

C) Beim interkostalen TAP Block wird das Lokalanästhetikum zwischen der Faszie des Musculus rectus abdominis und der Faszie des Musculus transversus abdominis appliziert.

D) Zur Blockade der Nervi ilioinguinalis und iliohypogastricus wird der Schallkopf initial auf einer Verbindungslinie zwischen der Spina iliaca anterior superior und dem Umbilicus aufgesetzt.

E) Der Nervus saphenus ist ein sensibler Ast des Nervus femoralis und innerviert die Unterschenkelinnenseite.

6) Topographische Anatomie: Welche der folgenden Aussagen ist/sind korrekt??

A) Bei der Punktion der A. brachialis kann der Nervus radialis verletzt werden.

B) Bei der Punktion der A. ulnaris kann der N. ulnaris verletzt werden.

C) Bei der Punktion der V. jugularis interna ist die Stichrichtung egal.

D) Arterien und Venen können zuverlässig anhand ihrer Komprimierbarkeit unterschieden werden.

E) Paarig angelegte Blutgefäße werden häufig auch von Nerven begleitet

7) Hygiene: Welche der folgenden Aussagen ist/sind **nicht** korrekt?

A) Die Handlungsempfehlungen für die Hygiene bei ultraschallgestützten Punktionen können als S1-Leitlinie bei AWMF, auf der DGAI-homepage oder unter „Anästh. Intensiv. 2015; 56:34-40 „ nachgelesen werden.

B) Wegen der erhöhten Gefahr der Infektion bei Manipulation sollte die Punktionsstelle nur bei klinischem Verdacht auf eine Infektion inspiziert werden.

C) Bei der Anlage eines zur kontinuierlichen Applikation von Lokalanästhetika vorgesehenen Katheters ist ein steriler Kittel für den Behandler obligat.

D) Bei der Verwendung alkoholischer Händedesinfektionsmittel ist in der Regel eine Einwirkzeit von 30s ausreichend.

E) Auch bei der Anlage oberflächlicher single-shot-Applikationen von Lokalanästhetika können in Einzelfällen besondere Hygienemaßnahmen erforderlich sein.

8) Axillärer Plexus und Unterarmnerven: Welche der folgenden Aussagen ist/sind korrekt?

A) Der N. musculocutaneus begleitet die A. axillaris bis zum Ellenbogen.

B) Bei der Injektion des Lokalanästhetikums werden die oberflächlich liegenden Nerven zuerst betäubt.

C) Bei Patienten mit arterieller Verschlusskrankheit ist wegen der Gefahr der Gefäßkonstriktion die Indikation zu Nervenplexus-Anästhesie streng zu stellen.

D) Die Betäubung des axillären Nervenplexus kann für die Anlage einer Oberarmblutsperre nicht ausreichen.

E) Bei der Punktion in Kurz-Achsen-Technik ist das Risiko einer versehentlichen arteriellen Punktion gering.

9) Neuraxiale Blockaden: Welche der folgenden Aussagen trifft/treffen **nicht** zu?

A) Die Verwendung von Ultraschall kann bei Patienten mit hohem body-mass-index indiziert sein.
B) Der sympathische Grenzstrang kann beim Paravertebralblock nicht mitbetäubt werden.
C) Der Paravertebralblock hat ein geringeres Nebenwirkungsprofil als der Periduralkatheter.
D) Bei der ultraschallgestützten Paravertebralblock-Anlage kommt ein Konvexschallkopf mit niedriger Hz-Zahl zur Anwendung.
E) Der Paravertebralblock kann bei Bedarf auf mehreren Ebenen gelegt werden.

10) Der Plexus brachialis kommt in der Regel infraklavikulär:

A) Medial der A .subclavia
B) Dorsal der V. subclavia
C) Lateral der A. subclavia
D) Ventral der V. subclavia
E) Zwischen A. und V. subclavia

zu liegen.

11) Die typische Schallauslöschung beim Ultraschall des supraklavikulären Plexus hinter der A. subclavia kommt durch:

A) Die Pleura parietalis
B) Die Pleura visczeralis
C) Totalauslöschung bei Übergang flüssig-fest
D) Die Clavicula
E) Die erste Rippe

12) Folgende Aussage/n zur dopplersonographischen Untersuchung der Hirnbasisarterien ist/sind richtig:

A) Es empfiehlt sich die Untersuchung mit dem transtemporaler Zugangsweg zu beginnen.
B) Der Hauptstamm der A. cerebri media (M1-Segment) findet sich üblicherweise in einer Tiefe von 50-55 mm.
C) Die A. cerebri media weist ein Flußprofil auf, welches sich von der Sonde entfernt.
D) Eine Darstellung des Karotissiphons ist im transorbitalen Zugang nicht möglich.
E) Eine mittlere Flussgeschwindigkeit über der A. cerebri media von größer 60 cm/s wird als pathologisch angesehen.
F) Im transtemporalen Zugangsweg lassen sich die A. cerberi media, anterior und posterior, sowie die A. carotis interna ableiten.

13) Welche der folgenden Aussagen zur Venenthrombose ist/sind richtig?

1. Bei einer frischen Venenthrombose stellt sich das Lumen echroreich dar.
2. Ein randumspülter Thrombus ist Zeichen einer mehr als 10 Tage alten Thrombose.
3. Der Durchmesser einer frisch thrombosierten Vene ist in der Regel kleiner als der der korrespondierenden Arterie.
4. Zum sicheren Nachweis einer Venenthrombose ist aktuell eine farbkodierte Duplexsonographie erforderlich.
5. Bei einem Wells-Score > 2 kann auf die sonograpische Abklärung verzichtet werden.

A) 1 und 4 sind richtig.
B) Nur 2 ist richtig.
C) 3 und 5 sind richtig
D) Alle Aussagen sind falsch.
E) Alle Aussagen sind richtig.

14) Welche Aussage/n zum Thema Neurosonographie ist/sind richtig?

A) Bei streng orthogonaler Anschallung kann mittels Doppler Nerv von Gefäß unterschieden werden
B) Zur Darstellung von oberflächlichen Nerven eignet sich besonders gut ein niedriges Frequenzspektrum
C) Nervenstrukturen stellen sich im Ultraschallbild uniform dar
D) Nerven haben im Longitudinalschnitt eine parallel-fibrilläre Struktur und sind somit von Sehnen zu unterscheiden.
E) Nerven haben im Longitudinalschnitt eine plexiform-faszikuläre Struktur und sind somit von Sehnen zu unterscheiden.

15) Die Leitlinien zur interventionellen Sonografie des *European Federation of Societies for Ultrasound in Medicine and Biology* empfehlen Gefäßpunktionen durch direkte Ultraschall-Technik durchzuführen. Für die Anlage welcher Katheter bzw. Zugänge sollte dies laut Empfehlungen der EFSUMB in Betracht gezogen werden?

1. Katheterisierung der radialen Arterie
2. Peripher venöse Zugänge
3. Zentral venöse Zugänge der Vena jugularis interna
4. Zentral venöse Zugänge der Vena subclavia
5. Zentral venöse Zugänge derVena femoralis

A) 3 und 4 ist richtig
B) 1, 3 und 5 ist richtig
C) 1, 2 und 3 ist richtig
D) 1, 3, 4 und 5 ist richtig
E) 1, 2 und 4 ist richtig

Vielen Dank für Ihre Teilnahme.
