# Supplementary material for: Efficacy of a certified modular ultrasound curriculum
Source: Anaesthesist. 2020 Feb 13;69(3):192–7. doi: 10.1007/s00101-020-00730-9 (PMC7056694; doi:10.1007/s00101-020-00730-9)
Supplement: Supplementary file 2 — ESM_Kurs 2_Fragebogen/Questionnaire (in German) [file 101_2020_730_MOESM2_ESM.pdf]

INTERAKTIVES QUIZ ÜBER DIE AFS MODULE 4+5

AFS Modul 4 – (Transthorakale) Echokardiografie

AFS Modul 5 – Thorakoabdominelle Sonografie (E-FAST plus)

ANLEITUNG

Gleich werden Ultraschallbilder bzw. Videosequenzen gezeigt.

- Es sind Mehrfachantworten möglich.
- Bitte den ausgefüllten Antwortbogen ins Studienpostfach legen bzw. per Hauspost schicken.
- Herzlichen Dank fürs Mitmachen und viel Spaß!

FRAGE 1

Welche Aussagen zum nebenstehenden Video treffen zu?

A. Normalbefund

B. Leichtgradig eingeschränkte LV-EF (ca. 45%)

C. Mittelgradig eingeschränkte LV-EF (ca. 35%)

D. Hochgradig eingeschränkte LV-EF (ca. 20%)

E. Die LV-EF ist nicht beurteilbar.

FRAGE 2

Was ist auf dem Video zu erkennen?

A. Pleuraerguss

B. Perikarderguss mit deutlicher Kompression des RV

C. Perikarderguss ohne wesentliche hämodynamische Auswirkungen

D. Deutlich eingeschränkte RV-EF

E. Deutlich eingeschränkte LV-EF

FRAGE 3

Welche Aussagen zum nebenstehenden Video treffen zu?

A. Apikale 4-KB Anlotung

B. Parasternale lange Achse (long axis, LAX) Anlotung

C. Parasternale kurze Achse (short axis, SAX) Anlotung

D. Subcostale lange Achse (long axis, LAX) Anlotung

E. Subcostale kurze Achse (short axis, SAX) Anlotung

FRAGE 4

Welche Aussagen zum nebenstehenden Video treffen zu?

A. Parasternale LAX Anlotung

B. Parasternale SAX Anlotung

C. Subcostale Anlotung

D. Apikale Anlotung (4-KB)

E. Apikale Anlotung (2-KB)

FRAGE 5

Was ist im Video zu sehen?

A. Die luftgefüllte Lunge

B. Die atelektatische Lunge

C. Das Zwerchfell

D. Aszites

E. Ein Pleuraerguss

FRAGE 6

Was ist im unteren Teil des Bildes gezeigt?

A. B-Mode Bild

B. M-Mode Bild

C. Lungenpunkt

D. Lungenpuls

E. Ein physiologischer Befund

FRAGE 7

Welche Aussagen zum nebenstehenden Video treffen zu?

A. Normalbefund

B. Leichtgradig eingeschränkte LV-EF (45%)

C. Mittelgradig eingeschränkte LV-EF (35%)

D. Hochgradig eingeschränkte LV-EF (20%)

E. Die LV-EF ist nicht beurteilbar.

FRAGE 8

Welche Aussagen zum nebenstehenden Video treffen zu?

A. Normalbefund

B. Hypermobiler RV

C. Mittelgradig eingeschränkte LV-EF (35%)

D. Leichtgradige eingeschränkte LV-EF (45%)

E. Deutlich vergrößerter linker Vorhof

FRAGE 9

Was ist auf dem Video zu sehen?

A. Normalbefund

B. Mittelgradig eingeschränkte LV-EF

C. Zeichen einer Rechtsherzbelastung

D. Vermutlich Pathologie der Mitralklappe

E. Zeichen einer Lungenarterienembolie

FRAGE 10

Was ist im Video zu sehen?

A. Die Leber

B. Die Milz

C. Das sog. „Vorhangphänomen“

D. Ein Pleuraerguss

E. Ein physiologischer Befund

FRAGE 11

Was zeigt das nebenstehende Video?

A. Einen Normalbefund

B. V. cava inferior

C. Gestaute Lebervenen

D. Zeichen einer

Rechtsherzinsuffizienz

E. Langstreckiges Aortenaneurysma

FRAGE 12

Was ist im Video zu sehen?

A. Längs angelotete Rippen

B. Pleuragleiten

C. Pneumothorax

D. Lungenpuls

E. Ein pathologischer Befund

FRAGE 13

Was zeigt das nebenstehende Bild?

A. eine Niere mit Zysten

B. Harnstau bei Ablaufstörung im Bereich des Nierenbeckens

C. Harnstau bei Ablaufstörung im Bereich des proximalen Ureters

D. Harnstau bei Ablaufstörung im Bereich des distalen Ureters

E. einen Normalbefund

FRAGE 14

Was zeigt der untere Bereich des Bildes aus dem Bereich des Thorax?

A. M-Mode

B. B-Mode

C. Einen Lungenpuls

D. Einen Lungenpunkt

E. Das sog. „seashore"-Zeichen

FRAGE 15

Was ist auf dem Video zu erkennen?

A. Pleuraerguss

B. Perikarderguss mit deutlicher Kompression des RV

C. Perikarderguss ohne wesentliche hämodynamische Auswirkungen

D. Deutlich eingeschränkte RV-EF

E. Deutlich eingeschränkte LV-EF

FRAGE 16

Was ist auf dem Bild zu sehen?

A. Leber

B. Milz

C. Niere

D. Morison-Pouch

E. Koller-Pouch

FRAGE 17

Welche Aussagen zu dem Video treffen zu?

A. Normalbefund

B. Leichtgradig eingeschränkte LV-EF (45%)

C. Mittelgradig eingeschränkte LV-EF (35%)

D. Hochgradig eingeschränkte LV-EF (20%)

E. Die LV-EF ist nicht beurteilbar.

FRAGE 18

Welche Aussagen zum nebenstehenden Video treffen zu?

A. Normalbefund

B. Leichtgradig eingeschränkte LV-EF (ca. 45%)

C. Mittelgradig eingeschränkte LV-EF (ca. 30%)

D. Hochgradig eingeschränkte LV-EF (ca. 15%)

E. Die LV-EF ist nicht beurteilbar

FRAGE 19

Welche Aussagen zum nebenstehenden Video treffen zu?

A. Parasternale LAX Anlotung

B. Parasternale SAX Anlotung

C. Subcostale Anlotung

D. Apikale Anlotung (4-KB)

E. Apikale Anlotung (2-KB)

FRAGE 20

Was zeigen die nebenstehenden Bilder?

A. Einen Normalbefund

B. Dieselbe anatomische Struktur in zwei Anlotungen

C. Gestaute Lebervenen

D. Zeichen einer Rechtsherzinsuffizienz

E. Langstreckiges Aortenaneurysma
